# Population‐based cohort imaging: skeletal muscle mass by magnetic resonance imaging in correlation to bioelectrical‐impedance analysis

**DOI:** 10.1002/jcsm.12913

**Published:** 2022-01-25

**Authors:** Lena S. Kiefer, Jana Fabian, Susanne Rospleszcz, Roberto Lorbeer, Jürgen Machann, Mareen S. Kraus, Marc Fischer, Frank Roemer, Wolfgang Rathmann, Christa Meisinger, Margit Heier, Konstantin Nikolaou, Annette Peters, Corinna Storz, Christopher L. Schlett, Fabian Bamberg

**Affiliations:** ^1^ Department of Diagnostic and Interventional Radiology University of Tuebingen Tuebingen Germany; ^2^ Department of Epidemiology Ludwig‐Maximilians‐University München Munich Germany; ^3^ Institute of Epidemiology, Helmholtz Zentrum München German Research Center for Environmental Health Neuherberg Germany; ^4^ Department of Radiology Ludwig‐Maximilians‐University Hospital Munich Germany; ^5^ German Centre for Cardiovascular Research (DZHK e.V.) Munich Germany; ^6^ Section of Experimental Radiology, Department of Diagnostic and Interventional Radiology University of Tuebingen Tuebingen Germany; ^7^ Institute for Diabetes Research and Metabolic Diseases (IDM), Helmholtz Center Munich University of Tuebingen Tuebingen Germany; ^8^ German Center for Diabetes Research (DZD) Neuherberg Germany; ^9^ Department of Radiology University of Erlangen–Nuremberg Erlangen Germany; ^10^ Department of Radiology Boston University School of Medicine Boston MA USA; ^11^ Institute for Biometrics and Epidemiology German Diabetes Center Duesseldorf Germany; ^12^ Chair of Epidemiology Ludwig‐Maximilians‐University München, UNIKA‐T Augsburg Augsburg Germany; ^13^ Independent Research Group Clinical Epidemiology, Helmholtz Zentrum München German Research Center for Environmental Health Neuherberg Germany; ^14^ KORA Study Centre University Hospital Augsburg Augsburg Germany; ^15^ Department of Neuroradiology, University Medical Center Freiburg, Faculty of Medicine University of Freiburg Freiburg Germany; ^16^ Department of Diagnostic and Interventional Radiology, University Medical Center Freiburg, Faculty of Medicine University of Freiburg Freiburg Germany

**Keywords:** Skeletal muscle mass, Fat‐free skeletal muscle mass, Skeletal muscle segmentation, Magnetic resonance imaging, Quantitative imaging biomarker

## Abstract

**Background:**

Skeletal muscle mass is subjected to constant changes and is considered a good predictor for outcome in various diseases. Bioelectrical‐impedance analysis (BIA) and magnetic resonance imaging (MRI) are approved methodologies for its assessment. However, muscle mass estimations by BIA may be influenced by excess intramuscular lipids and adipose tissue in obesity. The objective of this study was to evaluate the feasibility of quantitative assessment of skeletal muscle mass by MRI as compared with BIA.

**Methods:**

Subjects from a population‐based cohort underwent BIA (50 kHz, 0.8 mA) and whole‐body MRI including chemical‐shift encoded MRI (six echo times). Abdominal muscle mass by MRI was quantified as total and fat‐free cross‐sectional area by a standardized manual segmentation‐algorithm and normalized to subjects' body height^2^ (abdominal muscle mass indices: AMMI_MRI_).

**Results:**

Among 335 included subjects (56.3 ± 9.1 years, 56.1% male), 95 (28.4%) were obese (BMI ≥ 30 kg/m^2^). MRI‐based and BIA‐based measures of muscle mass were strongly correlated, particularly in non‐obese subjects [*r* < 0.74 in non‐obese (*P* < 0.001) vs. *r* < 0.56 in obese (*P* < 0.001)]. Median AMMI_Total(MRI)_ was significantly higher in obese as compared with non‐obese subjects (3246.7 ± 606.1 mm^2^/m^2^ vs. 2839.0 ± 535.8 mm^2^/m^2^, *P* < 0.001, respectively), whereas the ratio AMMI_Fat‐free_/AMMI_Total_ (by MRI) was significantly higher in non‐obese individuals (59.3 ± 10.1% vs. 53.5 ± 10.6%, *P* < 0.001, respectively). No significant difference was found regarding AMMI_Fat‐free(MRI)_ (*P* = 0.424). In analyses adjusted for age and sex, impaired glucose tolerance and measures of obesity were significantly and positively associated with AMMI_Total(MRI)_ and significantly and inversely with the ratio AMMI_Fat‐free(MRI)_/AMMI_Total(MRI)_ (*P* < 0.001).

**Conclusions:**

MRI‐based assessment of muscle mass is feasible in population‐based imaging and strongly correlated with BIA. However, the observed weaker correlation in obese subjects may explain the known limitation of BIA in obesity and promote MRI‐based assessments. Thus, skeletal muscle mass parameters by MRI may serve as practical imaging biomarkers independent of subjects' body weight.

## Introduction

Skeletal muscle, as one of the largest body compartments in adults, has a fundamental influence on health. It determines the physical condition, provides mobility and plays a key role in hormone and glucose metabolism and homeostasis. Therefore, skeletal muscle is a cardinal phenotypic marker and may serve as a good predictor for outcome in numerous health and disease states as well as an ideal target for health preservation and/or improvement.^1^, ^2^

Skeletal muscle is subject to significant changes in mass, composition and function with ageing and in various diseases.^3^ These changes of morphology and physiology are based on inflammation, hormonal dysregulation, denervation of motor units, and intramuscular lipid deposition.^4^ Major consequences are not only mobility limitations and physical disability with an increased risk of falls, injuries, and hospitalization but also cardiometabolic disorders with consecutively increased adverse health‐events and all‐cause mortality.^1^, ^2^, ^5^ Due to ongoing demographic transition with progressive ageing and weight gain of the population, disease‐related and age‐related deterioration of skeletal muscle is an important public health issue.^4^ Thus, simple, practicable and valid research, screening, and diagnosing tools as well as robust biomarkers for the assessment of relevant muscle properties are of high clinical importance.

Currently, bioelectrical‐impedance analysis (BIA) is a commonly used methodology for body composition estimations and the rough assessment of skeletal muscle mass. Its strength is based on the easy and safe way to use and also on its cost‐efficiency compared with other methods. However, BIA features a large individual prediction error for the estimation of skeletal muscle mass, because its reliability is influenced by multiple factors [e.g. device‐related factors, such as intra‐instrumental/inter‐instrumental variability), operator‐related factors (such as intra‐operator/inter‐operator variability), subject‐related factors (such as age, ethnicity, body weight and temperature, fasting and hydration state, and recent activity), and environment‐related factors (such as room temperature)]. Also, BIA is not able to accurately determine intramuscular fatty and fibrous components.^6^ In contrast, magnetic resonance imaging (MRI) with its lack of ionizing radiation, excellent soft tissue contrast, and high spatial resolution provides the possibility of simultaneous assessment of mass and other morphological and textural features and is therefore particularly suited with regard to large cohort settings. Furthermore, it is also sensitive to very small changes in muscle mass and allows concomitant evaluation of surrounding structures relevant for muscle activity and performance, such as bones, joints, or tendons.^6^, ^7^

Therefore, our hypothesis is that MRI is a feasible alternative to other accepted modalities of skeletal muscle assessment and that muscle mass parameters as assessed by MRI may serve as feasible imaging biomarkers in clinical and research settings independent of subjects' body weight. Aim of the present study was to evaluate the feasibility of quantitative assessment of total and fat‐free muscle mass by MRI using a standardized manual segmentation approach in non‐obese and obese subjects from the general population. This segmentation approach allows for the simultaneous assessment of additional muscle parameters (such as total, intramyocellular, and extramyocellular fat content), complementing quantitative and qualitative analyses.

## Materials and methods

### Study design and population

Subjects were derived from the KORA‐FF4 study (2013–2014, *n* = 2279), the second follow‐up of a population‐based survey within the Cooperative Health Research in the Region of Augsburg (KORA) survey. The design of the KORA studies has been described in detail previously.^8^ In brief, a comprehensive health assessment was prospectively performed for all subjects. Additionally, 400 subjects underwent whole‐body MRI according to previously described inclusion and exclusion criteria.^8^ Median time to MRI examination was 33 days (IQR: 24–45 days) after the health assessment. The study was approved by the ethics committee of the Bavarian Chamber of Physicians, Munich, Germany, and the local institutional review board of the Ludwig‐Maximilians University Munich, Germany. The study complies with the Declaration of Helsinki, including written informed consent of all participants.

### Anthropometry

The body mass index (BMI) was calculated as weight in kg divided by body height squared in square meters, with body weight and height both measured at the study centre. Waist circumference was measured at the smallest abdominal circumference or, in obese subjects, in the midpoint of the lowest rib and the upper margin of the iliac crest. Hip circumference was determined at the most protruding part of the hips to the nearest 1 mm. Obesity was defined according to the WHO‐definition with a BMI of 30 kg/m^2^ as cut‐off value.^9^

### Bioelectrical‐impedance analysis

Whole‐body BIA‐scans were acquired using a body impedance analyser (BIA 2000‐S, Data‐Input, Pöcking, Germany) with an operating frequency of 50 kHz at 0.8 mA to determine total body fat mass, lean body mass, and appendicular muscle mass in kilograms as well as the correspondent indices in kg normalized to subjects' body height squared. Therefore, ohmic resistance was measured between the dominant hand wrist and dorsum and the dominant foot angle and dorsum in supine position.

Muscle mass in kg was then calculated by the following equation according to Janssen *et al*.^10^ and subsequently normalized to subjects' body height squared (skeletal muscle index: SMI_BIA_):

$$\mathrm{BIA}-\text{based skeletal muscle mass}\;\left(\mathrm{kg}\right)=\left(\text{body}\;{\text{height}}^{2}/\text{resistance}\times 0.401\right)+\left(\text{gender}\times 3.825\right)+\left(\mathrm{age}\times -0.071\right)+5.102$$

with body height in centimeter, resistance in Ω, for gender: male = 1 and female = 0 and age in years.^3^, ^10^

### Imaging protocol and data acquisition

Magnetic resonance imaging examinations were performed in supine position on a 3‐Tesla Magnetom Skyra (Siemens Healthineers, Erlangen, Germany) using an 18‐channel body surface coil in combination with a table‐mounted spine matrix coil. The complete imaging protocol and technical specificities have been described in detail elsewhere.^8^

For muscle segmentation, a T2*‐corrected, multi‐echo 3D‐gradient‐echo Dixon‐based sequence (multi‐echo Dixon) of the upper abdomen providing a coverage of approximately 40 to 50 cm with the following parameters was used (time to repetition (TR): 8.90 ms; time to echo (TEs): 1.23, 2.46, 3.69, 4.92, 6.15, and 7.38 ms; flip angle 4°, readout echo bandwidth 1080 Hz/pixel, matrix 256 × 256, slice thickness 4 mm). Data were acquired during a single breath‐hold of 15 s. The post‐processing algorithm using the Software MR LiverLab (Version VD13, Siemens Healthineers, Cary, USA) automatically calculated proton‐density fat‐fraction maps as DICOM‐files, which were used to simultaneously assess muscle mass and fat content.

### Image analysis and muscle segmentation

The DICOM‐files were implemented into the Software MITK (Version 2015.5.2, German Cancer Research Center, Heidelberg, Germany). Two independent observers blinded to any covariates performed muscle segmentation. Details of the applied segmentation approach have been described previously.^11^ In brief, each muscle compartment (both the right and left psoas major, quadratus lumborum, rectus abdominis, and autochthonous back muscles) was manually segmented according to standardized anatomical landmarks on one axial slice at the level of the lower endplate of the L3 vertebral body, because recent studies demonstrated that level L3 is a good surrogate for the entire lumbar spine and that skeletal muscle cross‐sectional area at this level is a reliable method for the determination of sarcopenia.^12^, ^13^ If L3 vertebra was not imaged, the most caudal possible axial slice was selected. Subjects with significant image artefacts on all levels were excluded from the analysis. The complete manual segmentation and post‐processing procedure took an average 10 min.

Interobserver and intraobserver reproducibility of the segmentation algorithm was assessed in a subset of 50 randomly selected subjects, being excellent for all included muscle compartments with only minor absolute and relative differences [intraclass correlation (ICC): 0.93–0.97, 31.0 ± 44.7 mm^2^, 2.7 ± 3.9%; ICC: 0.96–0.98, 5.5 ± 25.3 mm^2^, 0.5 ± 2.3%; respectively].^11^

### Total and fat‐free muscle mass

**Figure 1:**
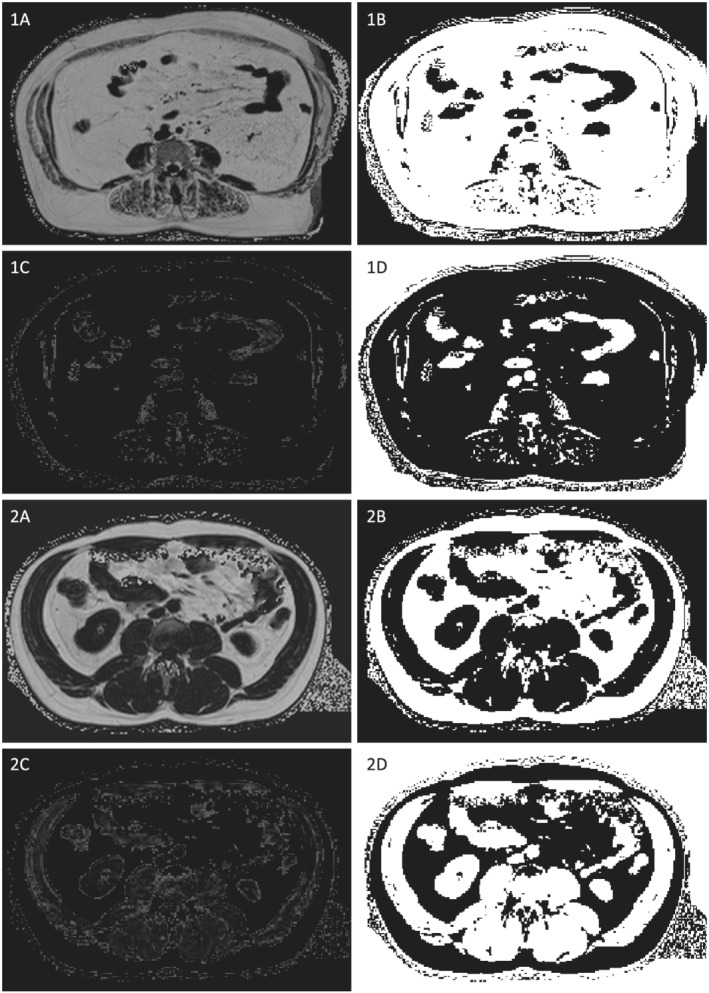
Example of an obese (A) and normal weight (B) subject with lower (A) and higher (B) total and fat‐free abdominal skeletal muscle mass as cross‐sectional areas by MRI (post‐processed with Matlab_R2017a). (1) Original proton‐density fat‐fraction map. (2) Segmentation of adipose tissue. (3) Segmentation of intra (myo)cellular lipids. (4) Segmentation of fat‐free muscle mass.

Muscle compartments were segmented as described earlier using dedicated, anatomical landmarks defining the exact muscle boundaries. Muscle cross‐sectional area (CSA) in mm^2^ was then used as surrogate for MRI‐based muscle mass. Total mass was defined as the complete muscle CSA_Total_ comprised by its muscle fascia, including macroscopically visible, intermyocellular‐intrafascial fatty septa. For the determination of fat‐free muscle mass (CSA_Fat‐free_), the segmented compartments were post‐processed using a semi‐automated, in‐house application (Matlab_R2017a, The MathWorks, Inc., Massachusetts, USA) (*Figure* *1*). Given the presumptions that even myocytes with a high amount of intramyocellular lipids do not feature an intensity value greater than approximately 200 (corresponding to 20% fat content) in proton‐density fat‐fraction maps and that every voxel with an intensity value greater than 200 therefore contains extramyocellular adipose tissue, a former approved threshold value of 200 was set to quantify those voxels that solely comprise myocytes with intramyocellular lipids, excluding extramyocellular, adipose tissue.^14^ Fat‐free mass was accordingly calculated by the following equation:

$${\mathrm{CSA}}_{\mathrm{Fat}-\text{free}}\;\left({\mathrm{mm}}^{2}\right)={\mathrm{CSA}}_{\text{Total}}\times \left(\text{intensity}\;{\text{value}}_{\text{Threshold}200}/\text{intensity}\;{\text{value}}_{\text{Threshold}1000}\right)$$

Total and fat‐free muscle mass were then normalized to subjects' body height squared calculating the correspondent indices in mm^2^/m^2^ indicated as total and fat‐free abdominal muscle mass index (AMMI_Total_ and AMMI_Fat‐free_, respectively).^15^ Furthermore, the ratio AMMI_Fat‐free_/AMMI_Total_ in % was calculated to indicate the amount of functionally contractable muscle tissue within the whole muscle compartments.

### Myosteatosis

Total myosteatosis was quantified as mean proton‐density fat‐fraction in percentage (PDFF_muscle_) in the same segmented muscle compartments at level L3 vertebra.^16^

### Visceral and subcutaneous adipose tissue

Visceral and subcutaneous adipose tissue (VAT and SAT, respectively) as abdominal adipose tissue compartments were segmented and quantified in square centimeters by a semi‐automated algorithm based on fuzzy‐clustering on one axial slice at the level of the umbilicus.^17^, ^18^

### Other covariates

To determine the glycaemic status, a 75 g oral glucose tolerance test was performed for all subjects not yet diagnosed with diabetes mellitus. According to the WHO definition, subjects were classified with an impaired glucose metabolism either with established type 2 diabetes mellitus (T2DM) or prediabetes and as healthy controls. Hypertension was determined as systolic blood pressure ≥140 mmHg and/or diastolic blood pressure ≥90 mmHg or current intake of antihypertensive medication, given that the subjects were aware of being hypertensive. Alcohol consumption and smoking status were classified by self‐report as no (0 g/day) or any alcohol consumption (≥0.1 g/day) and never‐smoker and ex‐smoker or current (regular/sporadic) smoker. Nutritional supply was evaluated with a 24 h food list and food frequency questionnaire. Regarding physical activity, subjects were categorized as physically active (regular physical activity ≥1 h/week) or physically inactive (irregular physical activity <1 h/week, almost no or no physical activity). Routinely intake of medication was generally categorized according to most recent guidelines. Standardized laboratory tests were used to determine important blood values. Dyslipidaemic changes of blood lipids were defined as increased levels of triglycerides, total cholesterol and LDL and decreased levels of HDL. The presence of somatic (musculoskeletal) symptoms, such as back pain and pain in arms, legs or joints was recorded in addition.

### Statistical analysis

Demographics and characteristics of the study population are presented as arithmetic mean ± standard deviation or median with 1st and 3rd quartile for continuous variables and absolute counts with percentages for categorical variables. Differences in mean values or counts between non‐obese and obese subjects were assessed by *t* test (quantitative data) or *χ* ^2^‐test (qualitative data). Correlations of MRI‐based and BIA‐based measurements of muscle mass as well as with demographics were evaluated by scatter plots and Pearson's correlation coefficients. Explorative associations of MRI‐based measurements of muscle mass with demographics and measures of obesity and adipose tissue were determined by linear regression adjusted for age and gender. Outcomes and covariates were standardized by subtracting the mean and dividing by standard deviation. Statistical significance was indicated by *P* values <0.05. Statistical analysis was performed using R V3.4.1 (R Core Team, www.r‐project.org, 2017).

## Results

### Study population

Among 400 subjects who underwent whole‐body MRI, 335 subjects (83.7%) were included in this analysis. Fifty‐seven subjects (14.3%) were excluded due to insufficient image quality (e.g. fat‐water swapping artefacts due to field inhomogeneities, and motion/breathing artefacts) or because they did not complete the imaging protocol and eight subjects (2%) were subsequently excluded due to missing values in any of the covariates. Demographics and characteristics of the study population are provided in *Table* *1*.

**Table 1:** Demographics of the study population

Characteristics	All subjects	Normal weight (BMI ≤ 30 kg/m^2^)	Obesity (BMI ≥ 30 kg/m^2^)	*P* value
	*N* = 335	*N* = 240 (71.6%)	*N* = 95 (28.4%)	
Age (years)	56.3 ± 9.1	55.8 ± 9.1	57.7 ± 9.1	0.100
Sex (male gender)	188 (56.1%)	136 (56.7%)	52 (54.7%)	0.84
Impaired glucose metabolism (prediabetes & T2DM)	127 (37.9%)	69 (28.8%)	58 (61.1%)	<0.001
Hypertension	110 (32.8%)	64 (26.7%)	46 (48.4%)	<0.001
Alcohol consumption (≥0.1 g/day)	253 (75.5%)	190 (79.2%)	63 (66.3%)	0.04
Current smoking status (regular or sporadic)	204 (60.9%)	138 (57.5%)	66 (69.5%)	0.005
HbA1c (%)	5.6 ± 0.8	5.5 ± 0.7	5.7 ± 0.8	0.022
Fasting serum glucose (mg/dL)	104.6 ± 23.3	102.0 ± 23.8	111.2 ± 20.6	0.001
Triglyceride levels (mg/dL)	130.3 ± 87.5	120.0 ± 86.9	156.5 ± 83.8	<0.001
Total cholesterol (mg/dL)	217.9 ± 36.2	218.4 ± 36.3	216.6 ± 36.4	0.69
HDL (mg/dL)	62.5 ± 17.9	65.3 ± 18.5	55.5 ± 14.1	<0.001
LDL (mg/dL)	139.2 ± 32.9	139.0 ± 33.3	139.9 ± 32.1	0.83
Vitamin D (calciferol)^†^ (ng/mL)	23.7 ± 11.9	24.4 ± 11.9	21.8 ± 11.7	0.07
Creatinine (mg/dL)	0.9 ± 0.2	0.9 ± 0.2	0.9 ± 0.1	0.21
Potassium (mmol/L)	4.3 ± 0.3	4.3 ± 0.3	4.3 ± 0.3	0.75
Nutrient supply^†^				
Energy (kcal/day)	1827.6 ± 408.3	1848.3 ± 405.7	1772.5 ± 412.9	0.18
Protein (mg/day)	69.8 ± 15.0	69.4 ± 15.0	70.8 ± 15.1	0.48
Physically active	204 (60.9%)	155 (64.6%)	49 (51.6%)	0.04
Medication				
Lipid‐lowering medication	35 (10.4%)	20 (8.3%)	15 (15.8%)	0.07
Non‐steroidal anti‐inflammatory drugs	10 (3.0%)	5 (2.1%)	5 (5.3%)	0.24
Oral antihyperglycaemic agents	28 (8.4%)	17 (7.1%)	11 (11.6%)	0.26
Musculoskeletal symptoms (pain in back, joints, arms, and legs)	128 (38.2%)	80 (33.3%)	48 (50.5%)	0.005

^†^ Based on *N* = 260.

In general, included subjects were predominantly middle‐aged men (mean age: 56.3 ± 9.1 years; male gender: 56.1%) with 95 subjects (28.4%) being classified as obese based on a BMI ≥ 30 kg/m^2^. Overall, obese subjects featured a more distinct cardiometabolic risk profile having a higher prevalence of impaired glucose metabolism, hypertension, and dyslipidaemia and being significantly less physically active. Furthermore, obese subjects had significantly lower levels of vitamin D, significantly higher consumption rates of alcohol and nicotine and a significantly higher prevalence of musculoskeletal pain symptoms (all *P* < 0.04; *Table* *1*).

### Body composition and muscle mass

Detailed measurements of body composition by anthropometry, BIA, and MRI are shown in *Table* *2*. Non‐obese subjects had a significantly lower waist and hip circumference in anthropometry and based on BIA significantly lower indices of total body fat mass, lean body, and appendicular muscle mass as well as calculated SMI_BIA_ (all *P* < 0.001).

**Table 2:** Anthropometric, BIA‐based and MRI‐based measurements of body composition

Measurements of body composition		All subjects	Normal Weight (BMI ≤ 30 kg/m^2^)	Obesity (BMI ≥ 30 kg/m^2^)	*P* value
		*N* = 335	*N* = 240 (71.6%)	*N* = 95 (28.4%)	
Anthropometry	Body height (m)	171.5 ± 9.7	172.4 ± 9.6	169.3 ± 9.4	0.01
	Body weight (kg)	81.9 ± 15.7	76.1 ± 12.6	96.6 ± 13.1	<0.001
	BMI (kg/m^2^)	27.8 ± 4.7	25.5 ± 2.7	33.7 ± 3.3	<0.001
	Waist circumference (cm)	97.6 ± 13.6	92.2 ± 10.9	111.2 ± 9.4	<0.001
	Hip circumference (cm)	106.3 ± 8.6	102.7 ± 5.6	115.6 ± 7.8	<0.001
BIA	Total body fat mass index (kg/m^2^)	9.1 ± 3.2	7.6 ± 1.9	12.8 ± 3.0	<0.001
	Lean body mass index (kg/m^2^)	18.7 ± 2.4	17.8 ± 2.1	20.9 ± 1.9	<0.001
	Appendicular muscle mass index (kg/m^2^)	7.8 ± 1.2	7.4 ± 1.1	8.8 ± 1.0	<0.001
	Skeletal muscle mass index (SMI_BIA_ in kg/m^2^)^‡^	9.2 ± 1.6	8.9 ± 1.5	10.0 ± 1.5	<0.001
MRI	Total abdominal skeletal muscle mass (CSA_Total_ in mm^2^)	8759.2 ± 2143.1	8514.9 ± 2080.8	9376.4 ± 2184.8	0.001
	AMMI_Total_ (mm^2^/m^2^)	2954.6 ± 585.4	2839.0 ± 535.8	3246.7 ± 606.1	<0.001
	Female	2625.1 ± 484.2	2495.3 ± 389.6	2939.0 ± 547.9	<0.001^†^
	Male	3212.2 ± 526.2	3101.8 ± 481.5	3501.1 ± 532.9	<0.001^†^
	Fat‐free abdominal skeletal muscle mass (CSA_Fat‐free_ in mm^2^)	5099.8 ± 1749.2	5107.8 ± 1705.4	5079.6 ± 1864.7	0.90
	AMMI_Fat‐free_ (mm^2^/m^2^)	1709.5 ± 492.5	1695.9 ± 478.3	1743.7 ± 527.8	0.42
	Female	1428.1 ± 344.5	1417.3 ± 367.8	1454.4 ± 282.5	1^†^
	Male	1929.5 ± 479.2	1909.0 ± 442.9	1983.0 ± 564.5	1^†^
	Ratio AMMI_Fat‐free_/AMMI_Total_ (%)	57.7 ± 10.6	59.3 ± 10.1	53.5 ± 10.6	<0.001
	Female	54.7 ± 10.0	56.6 ± 10.1	50.0 ± 8.4	<0.001^†^
	Male	60.0 ± 10.4	61.4 ± 9.7	56.3 ± 11.4	0.007^†^
	PDFF_muscle_ (%)	11.5 ± 4.7	10.8 ± 4.3	13.3 ± 5.2	<0.001
	VAT (cm^2^)	146.8 ± 84.7	122.6 ± 76.4	208.0 ± 73.4	<0.001
	SAT (cm^2^)	277.2 ± 116.6	229.5 ± 74.2	397.7 ± 117.7	<0.001

AMMI, abdominal muscle mass index; BIA, bioelectrical‐impedance analysis; BMI, body mass index; CSA, cross‐sectional area; MRI, magnetic resonance imaging; PDFF, proton‐density fat‐fraction; SAT, subcutaneous adipose tissue; VAT, visceral adipose tissue.

^†^ Bonferroni‐adjusted for three independent tests.

^‡^ Skeletal muscle mass index derived by the following equation and normalized to subjects body height squared: Skeletal muscle mass (kg) = (body height^2^/resistance × 0.401) + (gender × 3.825) + (age × −0.071) + 5.102 (body height in cm, resistance in Ω, for gender: male = 1 and female = 0, age is in years).

**Figure 2:**
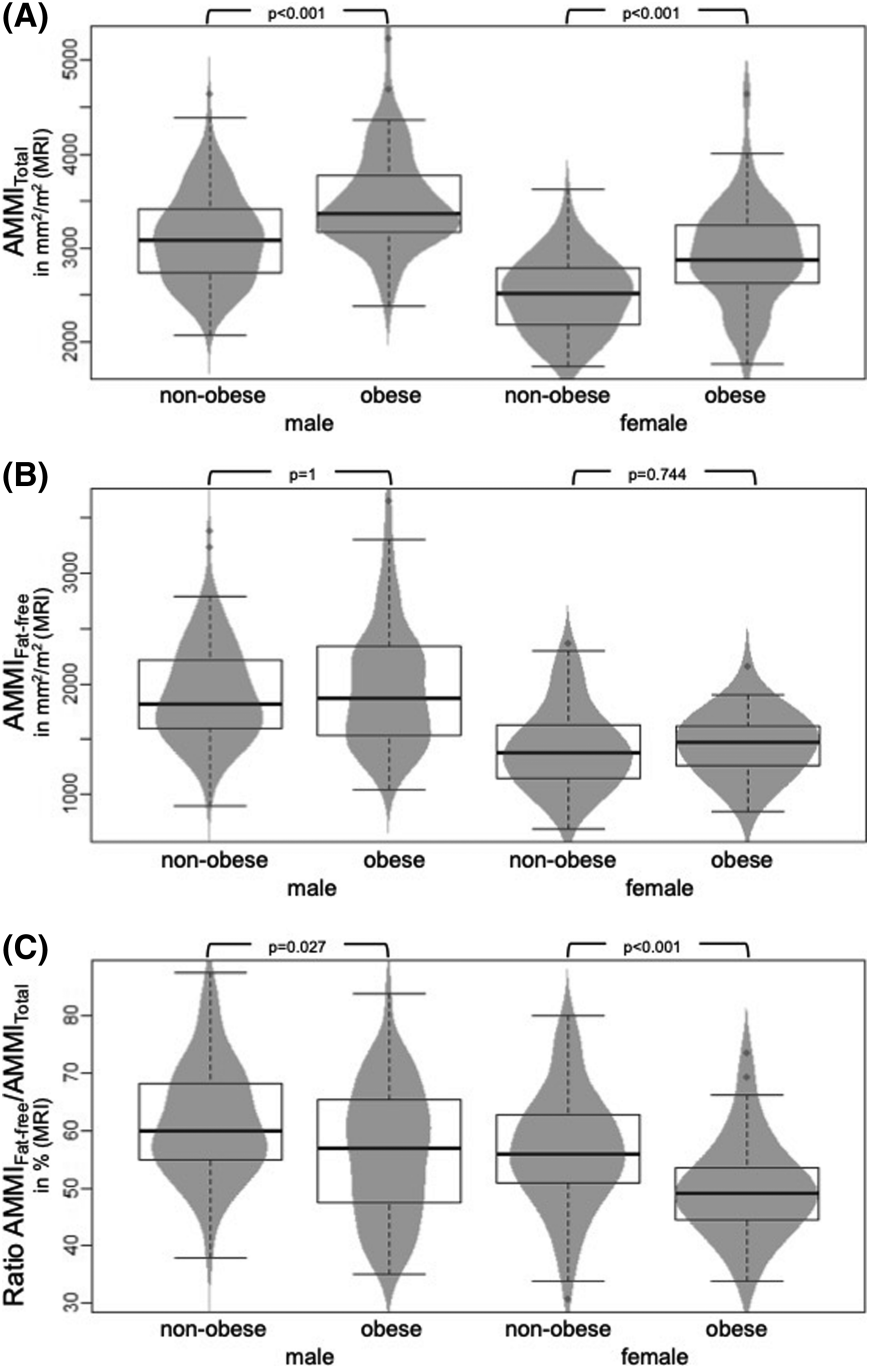
Differences in total abdominal muscle mass index (AMMI_Total)_ (A), fat‐free abdominal muscle mass index (AMMI_Fat‐free_) (B) and the ratio AMMI_Fat‐free_/AMMI_Total_ (C) between non‐obese and obese subjects and male and female subjects.

Based on MRI, mean total and fat‐free abdominal skeletal muscle mass of the entire sample by MRI was 8759.2 ± 2143 mm^2^ and 5099.8 ± 1749.2 mm^2^, respectively. AMMI_Total_ was lowest in female subjects with normal body weight and highest in obese male subjects (non‐obese female subjects: 2495.3 ± 389.6 mm^2^/m^2^, obese male subjects: 3501.1 ± 532.9 mm^2^/m^2^, *P* < 0.001). No significant difference was found regarding AMMI_Fat‐free_ between non‐obese and obese subjects (1695.9 ± 478.3 mm^2^/m^2^, 1743.7 ± 527.8 mm^2^/m^2^, respectively, *P* = 0.424). Concerning the ratio of normalized fat‐free to total abdominal skeletal muscle mass AMMI_Fat‐free_/AMMI_Total_ and in contrast to AMMI_Total_, normal weight male and female subjects had a significantly higher portion of fat‐free muscle tissue compared with obese, male and female subjects (male: 61.4 ± 9.7% and 56.3 ± 11.4%, *P* = 0.007, female: 56.6 ± 10.1% and 50.0 ± 8.4%, *P* < 0.001) (*Figure* 2). Furthermore, regarding the main body fat compartments by MRI, obese subjects with a BMI ≥ 30 kg/m^2^ showed significantly higher amounts of VAT, SAT, and PDFF_muscle_ compared with normal weights (*P* < 0.001).

### Correlation to bioelectrical‐impedance analysis

**Figure 3:**
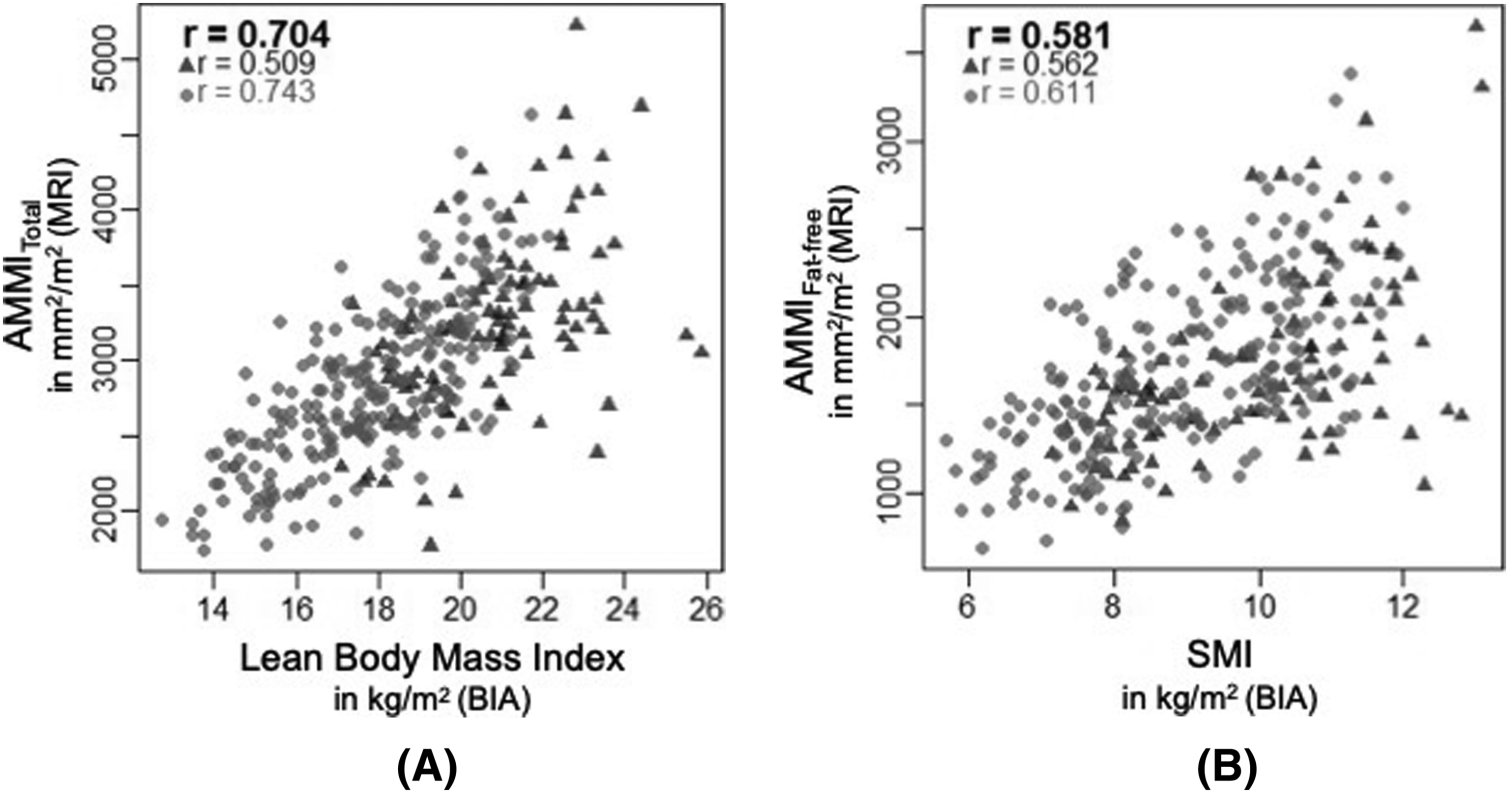
Correlations of magnetic resonance imaging (MRI)‐based and bioelectrical‐impedance analysis (BIA)‐based measurements of skeletal muscle mass in non‐obese (light circle) and obese (dark triangle) subjects.

Correlations of AMMI_Total_ and AMMI_Fat‐free_ with anthropometric and BIA‐based measurements are provided in *Table* 3, *Figures* 3 and S1. AMMI_Total_ and lean body mass index showed a strong and positive, linear correlation (*r* = 0.70), especially in normal weight subjects (*r* = 0.74). In contrast, AMMI_Fat‐free_ showed best correlation with calculated SMI_BIA_ with a moderate relationship (*r* = 0.58). In general, measures of muscle mass by MRI and BIA correlated considerably stronger in non‐obese compared with obese subjects (normal weight: *r* = 0.59–0.74, obesity: *r* = 0.47–0.56). After adjustment for age and sex, both AMMI_Total_ and AMMI_Fat‐free_ were significantly and positively associated with calculated SMI_BIA_ {β: 0.80 [95% confidence interval (CI): 0.66–0.94], *P* < 0.001; β: 0.42 (95% CI: 0.27–0.57), respectively, *P* < 0.001}. In further obesity‐stratified analyses, the age‐ and sex‐adjusted associations of MRI‐ and BIA‐based measurements of muscle mass were substantially weaker in obese individuals compared with normal weight subjects [association of calculated SMI_BIA_ with AMMI_Total_ in obese subjects: β: 0.50 (95% CI: 0.12–0.88), *P* < 0.011; with AMMI_Total_ in normal weight subjects: β: 0.77 (95% CI: 0.59–0.95), *P* < 0.001]; with AMMI_Fat‐free_ in obese subjects: β: 0.45 (95% CI: 0.08–0.83), *P* < 0.017; and with AMMI_Fat‐free_ in normal weight subjects: β: 0.53 (95% CI: 0.32–0.73), *P* < 0.001; respectively) (*Table* S2).

**Table 3:** Correlations of AMMI_Total_ and AMMI_Fat‐free_ with anthropometric and BIA‐based measurements of body composition

Measurements of body composition		Unadjusted			Adjusted for age and gender		
MRI	BIA and anthropometry	Estimate (Beta)	95% CI	*P* value	Estimate (Beta)	95% CI	*P* value
Total abdominal skeletal muscle mass index (AMMI_Total_)	Lean body mass index (kg/m^2^)	0.70	[0.63, 0.78]	<0.001	0.64	[0.54, 0.73]	<0.001
	Appendicular muscle mass index (kg/m^2^)	0.70	[0.62, 0.78]	<0.001	0.67	[0.56, 0.77]	<0.001
	Skeletal muscle mass index (SMI_BIA_ in kg/m^2^)^†^	0.67	[0.59, 0.75]	<0.001	0.80	[0.66, 0.94]	<0.001
	BMI (kg/m^2^)	0.47	[0.38, 0.57]	<0.001	0.44	[0.36, 0.52]	<0.001
	Hip circumference (cm)	0.28	[0.18, 0.38]	<0.001	0.28	[0.19, 0.37]	<0.001
	Waist circumference (cm)	0.53	[0.43, 0.62]	<0.001	0.40	[0.31, 0.50]	<0.001
Fat‐free abdominal skeletal muscle mass index (AMMI_Fat‐free_)	Lean body mass index (kg/m^2^)	0.48	[0.39, 0.58]	<0.001	0.28	[0.17, 0.38]	<0.001
	Appendicular muscle mass index (kg/m^2^)	0.52	[0.42, 0.61]	<0.001	0.29	[0.18, 0.41]	<0.001
	Skeletal muscle mass index (SMI_BIA_ in kg/m^2^)^†^	0.58	[0.49, 0.67]	<0.001	0.42	[0.27, 0.57]	<0.001
	BMI (kg/m^2^)	0.13	[0.02, 0.23]	0.02	0.11	[0.02, 0.20]	0.02
	Hip circumference (cm)	0.03	[−0.08, 0.14]	0.58	0.03	[−0.06, 0.12]	0.55
	Waist circumference (cm)	0.19	[0.09, 0.30]	<0.001	0.02	[−0.08, 0.12]	0.71

AMMI, abdominal muscle mass index; BIA, bioelectrical‐impedance analysis; BMI, body mass index; CSA, cross‐sectional area; MRI, magnetic resonance imaging; PDFF, proton‐density fat‐fraction; SAT, subcutaneous adipose tissue; VAT, visceral adipose tissue.

Estimates are based on linear regression with standardized outcome and covariates. Standardization consisted of subtracting the mean and dividing by the standard deviation.

^†^ Skeletal muscle mass index derived by the following equation and normalized to subjects body height squared: Skeletal muscle mass (kg) = (body height^2^/resistance × 0.401) + (gender × 3.825) + (age × −0.071) + 5.102 (body height in cm, resistance in Ω, for gender: male = 1 and female = 0, age is in years).

### Predictors of muscle mass

Associations of muscle mass by MRI with demographics and cardiometabolic risk factors are provided in *Table* S1 and *Figure* S2. Regarding muscle composition, higher levels of PDFF_muscle_ indicating myosteatosis are associated with a significantly lower AMMI_Fat‐free_ and ratio AMMI_Fat‐free_/AMMI_Total_, respectively (*P* < 0.001).

In explorative analyses adjusted for age and gender, impaired glucose metabolism, measures of dyslipidaemia, obesity, and adipose tissue (elevated triglyceride levels, BMI ≥ 30 kg/m^2^, higher waist and hip circumference, VAT and SAT) as well as hypertension were significantly and positively associated with AMMI_Total_ (*P* < 0.01). Furthermore, a significantly positive association was found for AMMI_Total_ and musculoskeletal pain symptoms (*P* = 0.002) as well as for AMMI_Fat‐free_ and total cholesterol, LDL and creatinine (*P* < 0.03). However, no significant association of muscle mass parameters by MRI with physical activity, nutrient supply, alcohol and nicotine consumption was found (*P* > 0.05).

## Discussion

Skeletal muscle has a fundamental influence on physical and metabolic health. Its characteristics may therefore serve as imaging biomarkers for the general and cardiometabolic health status for both clinical and research purposes. In this study, we evaluated a straight‐forward approach to total and fat‐free muscle mass by MRI complementing other, qualitative imaging parameters of muscle composition, and compared it to one of the standard methods used for muscle quantification, that is, BIA. On the one hand, our results confirm the significant prediction error of muscle mass by BIA in obesity, with considerably stronger correlations of MRI‐based and BIA‐based measures in normal weight subjects. On the other hand, our results indicate different patterns of MRI‐derived mass parameters between obese and non‐obese subjects as well as specific associations with risk factors for cardiometabolic disease. Thus, MRI allows for a comprehensive assessment of different morphologic characteristics of muscle mass biomarkers, for example, in cardiometabolic risk stratification, independent of subjects' body weight.

Currently, there are several modalities available for body composition analysis and estimation/quantification of muscle mass, relying on different technologies and assessing different aspects of skeletal musculature. However, the most commonly applied methods have substantial limitations and disadvantages. First, anthropometry, as an indirect modality for muscle mass quantification, is generally limited by its susceptibility to significant, individual and obesity‐related prediction errors with the tendency to overestimate muscle mass.^19^, ^20^ Accordingly, anthropometric and MRI‐based measures showed no to only weak correlations in this study. Second, dual‐energy X‐ray absorptiometry (DEXA), which some authors consider as the reference standard for quantification of muscle mass,^6^ is less sensitive to small changes compared with, for example, CT and MRI^21^, ^22^ and additionally based on ionizing radiation, thus limiting its feasibility. Third, cross‐sectional imaging, such as CT, is indeed characterized by a considerably smaller error in quantifying total and fat‐free skeletal muscle muss. However, the amount of ionizing radiation involved in whole‐body CT is limiting its utility in clinical/preventive and also research settings. Also, MRI as another cross‐sectional modality has several contraindications (e.g. metallic implants and claustrophobia), is more time consuming and costly compared with, for example, BIA and DEXA and requires a higher amount of technical expertise. Fourth, BIA lacks standardization of the technical procedure and its validity may be strongly influenced by different disease conditions, hydration status and physical activity in temporal relation with the measurement procedure. Thereby, BIA generally tends to overestimate lean body mass and consecutively underestimates total body fat mass in conditions with water retention, such as liver, renal, or heart failure, after exercise and most importantly in obese subjects.^6^, ^23^ In summary, there is no available technique that serves all the requirements for the precise and practical measurement of skeletal muscle mass until date. Each method has its own advantages and limitations, none is fully standardized, and therefore, none serves as gold standard at present.

In this study, measures of muscle mass assessed by MRI and BIA correlated considerably stronger in normal weight compared with obese subjects. Also, obese subjects featured comparatively higher values in BIA‐derived measurements of lean body and appendicular muscle mass as well as calculated skeletal muscle mass index. Independent of subjects' actual muscle mass, BIA generally overestimates lean body and therewith muscle mass in obesity. Furthermore, BIA is unable to determine intramuscular lipids and adipose tissue, which might account for a large fraction of non‐myocellular content in muscle tissue. According to this, significant differences were not found regarding AMMI_Fat‐free_ between obese and non‐obese subjects by MRI, whereas in BIA, obese subjects featured significantly higher muscle mass parameters. Overall, these results confirm the well‐known, significant prediction error of muscle mass parameters by BIA in obese subjects, which accounts primarily for the markedly lower correlation coefficients in subjects with a BMI ≥ 30 kg/m^2^. Thus, MRI might outperform BIA regarding quantification of skeletal muscle mass parameters specifically in obese subjects with a BMI ≥ 30 kg/m^2^, for example, in the assessment of sarcopenic obesity, an obese subtype with relatively low muscle mass.

Another advantage of MRI is the possibility to additionally perform a morphological and qualitative evaluation of muscle tissue complementing mass measurements, such as the assessment of intramyocellular and extramyocellular fat content and their distribution patterns as well as further, textural analyses. Because mass alone does not predict muscular performance sufficiently, MRI may be particularly suited for muscle composition analyses determining further aspects of related to its functional properties. Given the high likelihood of a relationship between MRI‐detectable muscle composition patterns, muscular performance and cardiometabolic disease, associations between these quantitative imaging findings with both clinical performance and outcome (e.g. in ageing and development of cardiometabolic disease) need to be further determined in large cohort studies.^24^

Some limitations of this study should be taken into account. First, we did not compare our results to DEXA, which is considered as the current gold standard by some authors. However, previous studies have demonstrated the validity and reproducibility of the standardized, muscle quantification by multi‐echo Dixon that we used in this study.^11^ Second, muscle performance does not depend solely on mass. Besides distinct associations of cardiometabolic risk factors and MRI‐derived measurements of muscle composition in this study, these imaging biomarkers should further be evaluated regarding their diagnostic and prognostic value and therefore rather be seen as a complement for conventional strength and endurance tests. Third, this study focused on the evaluation of muscle mass parameters by MRI and their correlation with BIA‐based measurements in both non‐obese and obese subjects. We did not interpret our results in reference to a standard population and did not aim to categorize subjects as sarcopenic or find a cut‐off value for sarcopenia in this population. However, as it is generally accepted that an absolute muscularity below the 5th percentile indicates sarcopenia,^25^ the applied approach should be further evaluated as a simple estimate of the quantitative index in this condition.

The assessment of muscle mass parameters by MRI based on a standardized segmentation algorithm is a feasible alternative to BIA in population‐based imaging. The weaker correlation of BIA and MRI in obese subjects may explain the known limitation of BIA in obesity and further promote MRI‐based assessments in large cohort settings in both non‐obese and specifically in obese subjects. AMMI_Fat‐free_ and AMMI_Total_ as well as their ratio as surrogates for muscle mass by MRI might serve as promising imaging biomarkers for the comprehensive assessment of skeletal muscle and whole‐body composition analysis as well as for a more accurate, cardiometabolic risk stratification, independent of subjects' body weight.

## Conflict of interest

The authors declare that they have no conflicts of interest.

## Supporting information


**Figure S1.** Correlations of MRI‐ and BIA‐based measurements of skeletal muscle mass in non‐obese (light circle) and obese (dark triangle) subjects.
**Figure S2.** Correlations of AMMITotal (A) and AMMIFat‐free (B) with age, BMI, VAT and physical activity.
**Table S1.** Associations between demographics, cardiometabolic risk factors and AMMI_Total_ and AMMI_Fat‐free_
**Table S2.** Obesity‐stratified associations of MRI‐ and BIA‐based measurements of skeletal muscle muss.
