# Supplementary material for: Population‐based cohort imaging: skeletal muscle mass by magnetic resonance imaging in correlation to bioelectrical‐impedance analysis
Source: J Cachexia Sarcopenia Muscle. 2022 Jan 25;13(2):976–86. doi: 10.1002/jcsm.12913 (PMC8977960; doi:10.1002/jcsm.12913)
Supplement: Supplementary file 1 — Figure S1. Correlations of MRI‐ and BIA‐based measurements of skeletal muscle mass in non‐obese (light circle) and obese (dark triangle) subjects. Figure S2. Correlations of AMMITotal (A) and AMMIFat‐free (B) with age, BMI, VAT and physical activity. Table S1. Associations between demographics, cardiometabolic risk factors and AMMITotal and AMMIFat‐free Table S2. Obesity‐stratified associations of MRI‐ and BIA‐based measurements of skeletal muscle muss. [file JCSM-13-976-s001.zip › JCSM_12913_Supplementary Material.docx]

**Supplementary Material**

**Supplementary Figure 1** Correlations of MRI- and BIA-based measurements of skeletal muscle mass in non-obese (light circle) and obese (dark triangle) subjects.

**Supplementary Figure 2** Correlations of AMMI_Total_ **(A)** and AMMI_Fat-free_ **(B)** with age, BMI, VAT and physical activity.

Light circle: non-obese subjects; dark triangle: obese subjects

| Predictor | Estimate (Beta) | | | 95%-CI | | | p-value | | |  |
| --- | --- | --- | --- | --- | --- | --- | --- | --- | --- | --- |
|  | **AMMI_Total_** | **AMMI_Fat-free_** |  | **AMMI_Total_** | **AMMI_Fat-free_** |  | **AMMI_Total_** | **AMMI_Fat-free_** |  | |
| Impaired glucose tolerance (Prediabetes & T2DM) | 205.61 | -34.81 |  | [87.46, 323.75] | [-130.96, 61.34] |  | <0.001 | 0.48 |  | |
| BMI (kg/m^2^) | 258.31 | 53.64 |  | [210.83, 305.80] | [9.92, 97.35] |  | <0.001 | 0.02 |  | |
| Waist circumference (cm) | 235.31 | 9.14 |  | [179.85, 290.78] | [-39.69, 57.98] |  | <0.001 | 0.71 |  | |
| Hip circumference (cm) | 163.47 | 13.35 |  | [111.65, 215.30] | [-30.46, 57.15] |  | <0.001 | 0.55 |  | |
| Obesity (BMI ≥30kg/m^2^) | 429.47 | 83.77 |  | [316.88, 542.07] | [-13.30, 180.83] |  | <0.001 | 0.09 |  | |
| Alcohol consumption | -97.94 | -85.24 |  | [-226.46, 30.57] | [-188.05, 17.56] |  | 0.14 | 0.10 |  | |
| Smoking status (current, regular or sporadic) | 112.58 | 82.04 |  | [-38.37, 263.53] | [-38.86, 202.95] |  | 0.14 | 0.18 |  | |
| Hypertension | 155.39 | -7.37 |  | [31.20, 279.58] | [-107.68, 92.93] |  | 0.01 | 0.89 |  | |
| HbA1c (%) | 19.60 | -17.00 |  | [-36.40, 75.60] | [-61.82, 27.81] |  | 0.49 | 0.46 |  | |
| Fasting serum glucose (mg/dl) | 43.82 | -23.42 |  | [-13.08, 100.72] | [-69.06, 22.21] |  | 0.13 | 0.31 |  | |
| Triglyceride levels (mg/dl) | 89.55 | 17.62 |  | [33.29, 145.81] | [-28.03, 63.27] |  | 0.002 | 0.45 |  | |
| Total cholesterol (mg/dl) | 23.99 | 47.67 |  | [-31.37, 79.36] | [3.60, 91.73] |  | 0.40 | 0.03 |  | |
| HDL (mg/dl) | -136.78 | -52.33 |  | [-195.12, -78.44] | [-100.17, -4.49] |  | <0.001 | 0.03 |  | |
| LDL (mg/dl) | 34.87 | 54.49 |  | [-20.11, 89.86] | [10.77, 98.21] |  | 0.21 | 0.02 |  | |
| Vitamin D (Calciferol)* (ng/ml) | -30.81 | 23.87 |  | [-85.79, 24.17] | [-20.14, 67.88] |  | 0.27 | 0.29 |  | |
| Creatinine (mg/dl) | 57.75 | 78.24 |  | [-9.36, 124.86] | [24.96, 131.52] |  | 0.09 | 0.004 |  | |
| Potassium (mmol/l) | 55.05 | 6.36 |  | [-0.33, 110.43] | [-38.22, 50.93] |  | 0.05 | 0.78 |  | |
| Nutrient supply  Energy (kcal/day)  Protein (mg/day) | -36.80  30.63 | -33.56  -5.67 |  | [-112.60, 39.00]  [-39.30, 100.56] | [-94.05, 26.94]  [-61.59, 50.25] |  | 0.34  0.39 | 0.28  0.84 |  | |
| Physically active | -34.47 | 40.57 |  | [-146.98, 78.04] | [-49.42, 130.57] |  | 0.55 | 0.38 |  | |
| Medication  Lipid-lowering medication  Non-steroidal anti- inflammatory drugs  Oral antihyperglycemic agents | 90.99  60.76  -88.09 | -102.40  -158.18  -171.47 |  | [-92.04, 274.02]  [-266.25, 387.77]  [-289.26, 113.09] | [-248.69, 43.89]  [-419.42, 103.05]  [-331.61, -11.34] |  | 0.33  0.72  0.39 | 0.17  0.23  0.04 |  | |
| Musculoskeletal symptoms (pain in back, joints, arms & legs) | 173.22 | 96.58 |  | [61.87, 284.56] | [6.81, 186.35] |  | 0.002 | 0.04 |  | |
| VAT (cm^2^) | 193.30 | 10.85 |  | [131.24, 255.36] | [-41.56, 63.25] |  | <0.001 | 0.69 |  | |
| SAT (cm^2^) | 126.43 | -31.20 |  | [72.58, 180.28] | [-75.54, 13.14] |  | <0.001 | 0.17 |  | |
| PDFF_muscle_ (%) | 58.14 | -189.55 |  | [-6.36, 122.64] | [-237.21, -141.90] |  | 0.08 | <0.001 |  | |

**Supplementary Table 1** Associations between demographics, cardiometabolic risk factors and AMMI_Total_ and AMMI_Fat-free_.

β-coefficients derived from linear regression model adjusted for age and gender. All continuous covariates were standardized.

| **Obese** | | | | | **Normal Weight** | | | | |
| --- | --- | --- | --- | --- | --- | --- | --- | --- | --- |
|  | adjusted for age & sex | | | |  | adjusted for age & sex | | | |
|  |  | **Estimate (Beta)** | **95%-CI** | **p-value** |  |  | **Estimate (Beta)** | **95%-CI** | **p-value** |
|  |  |  |  |  |  |  |  |  |  |
| **Total Abdominal Skeletal Muscle Mass Index (AMMI_Total_)** | Lean Body Mass Index (kg/m^2^) | 0.49 | [0.16, 0.83] | 0.005 | **Total Abdominal Skeletal Muscle Mass Index (AMMI_Total_)** | Lean Body Mass Index (kg/m^2^) | 0.81 | [0.66, 0.95] | <0.001 |
|  | Appendicular Muscle Mass Index (kg/m^2^) | 0.48 | [0.12, 0.83] | 0.009 |  | Appendicular Muscle Mass Index (kg/m^2^) | 0.8 | [0.64, 0.95] | <0.001 |
|  | Skeletal muscle mass index (kg/m^2^)* | 0.5 | [0.12, 0.88] | 0.01 |  | Skeletal muscle mass index (kg/m^2^)* | 0.77 | [0.59, 0.95] | <0.001 |
| **Fat-free Abdominal Skeletal Muscle Mass Index (AMMI_Fat-free_)** | Lean Body Mass Index (kg/m^2^) | 0.28 | [-0.06, 0.61] | 0.11 | **Fat-free Abdominal Skeletal Muscle Mass Index (AMMI_Fat-free_)** | Lean Body Mass Index (kg/m^2^) | 0.51 | [0.34, 0.69] | <0.001 |
|  | Appendicular Muscle Mass Index (kg/m^2^) | 0.3 | [-0.05, 0.66] | 0.09 |  | Appendicular Muscle Mass Index (kg/m^2^) | 0.51 | [0.32, 0.70] | <0.001 |
|  | Skeletal muscle mass index (kg/m^2^)* | 0.45 | [0.08, 0.83] | 0.02 |  | Skeletal muscle mass index (kg/m^2^)* | 0.53 | [0.32, 0.73] | <0.001 |
| **Ratio AMMI_Fat-free_/AMMI_Total_** | Lean Body Mass Index (kg/m^2^) | -0.04 | [-0.38, 0.30] | 0.82 | **Ratio AMMI_Fat-free_/AMMI_Total_** | Lean Body Mass Index (kg/m^2^) | -0.1 | [-0.31, 0.11] | 0.34 |
|  | Appendicular Muscle Mass Index (kg/m^2^) | -0.01 | [-0.36, 0.35] | 0.98 |  | Appendicular Muscle Mass Index (kg/m^2^) | -0.1 | [-0.32, 0.12] | 0.39 |
|  | Skeletal muscle mass index (kg/m^2^)* | 0.14 | [-0.24, 0.52] | 0.46 |  | Skeletal muscle mass index (kg/m^2^)* | -0.04 | [-0.28, 0.20] | 0.75 |

**Supplementary Table 2** Obesity-stratified associations of MRI- and BIA-based measurements of skeletal muscle muss.

Estimates are based on linear regression with standardized outcome and covariates. Standardization consisted of subtracting the mean and dividing by the standard deviation.

* Skeletal muscle mass index derived by the following equation and normalized to subjects body height squared:

Skeletal muscle mass (kg) = (body height^2^/resistance x 0.401) + (gender x 3.825) + (age x -0.071) + 5.102

(body height in cm, resistance in Ω, for gender: male=1 and female=0, age is in years).
