# Supplementary figures and images for: Population‐based cohort imaging: skeletal muscle mass by magnetic resonance imaging in correlation to bioelectrical‐impedance analysis
Source: J Cachexia Sarcopenia Muscle. 2022 Jan 25;13(2):976–86. doi: 10.1002/jcsm.12913 (PMC8977960; doi:10.1002/jcsm.12913)

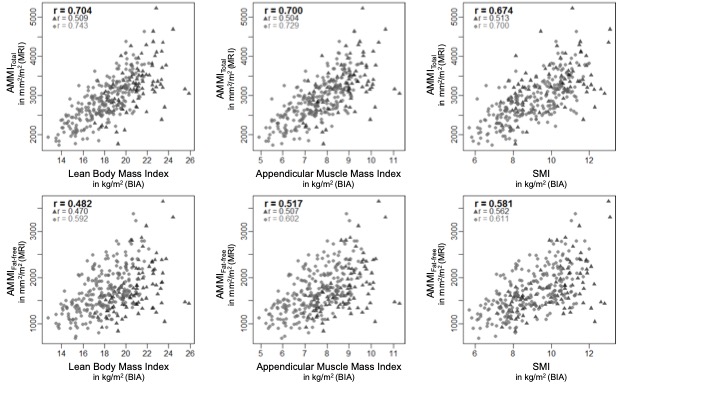

Supplement: Supplementary file 1 — Figure S1. Correlations of MRI‐ and BIA‐based measurements of skeletal muscle mass in non‐obese (light circle) and obese (dark triangle) subjects. Figure S2. Correlations of AMMITotal (A) and AMMIFat‐free (B) with age, BMI, VAT and physical activity. Table S1. Associations between demographics, cardiometabolic risk factors and AMMITotal and AMMIFat‐free Table S2. Obesity‐stratified associations of MRI‐ and BIA‐based measurements of skeletal muscle muss. [file JCSM-13-976-s001.zip › JCSM_12913_SuppFig1.jpeg]

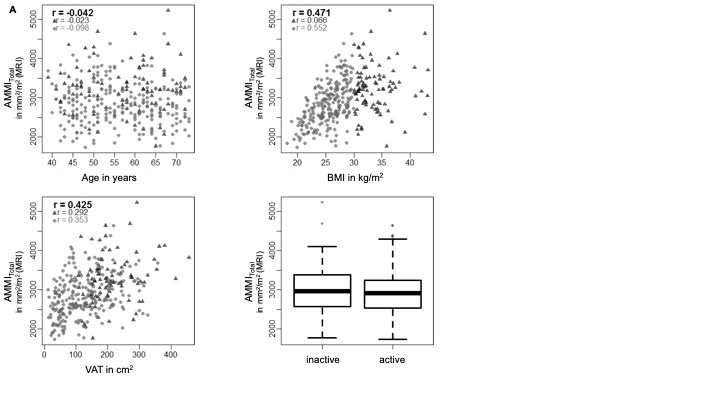

Supplement: Supplementary file 1 — Figure S1. Correlations of MRI‐ and BIA‐based measurements of skeletal muscle mass in non‐obese (light circle) and obese (dark triangle) subjects. Figure S2. Correlations of AMMITotal (A) and AMMIFat‐free (B) with age, BMI, VAT and physical activity. Table S1. Associations between demographics, cardiometabolic risk factors and AMMITotal and AMMIFat‐free Table S2. Obesity‐stratified associations of MRI‐ and BIA‐based measurements of skeletal muscle muss. [file JCSM-13-976-s001.zip › JCSM_12913_SuppFig2A.jpeg]

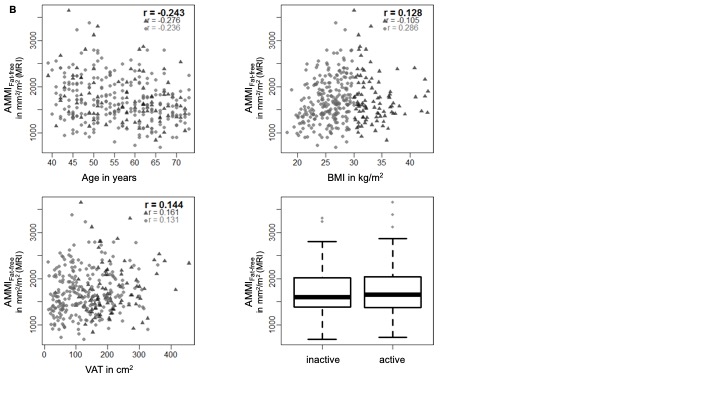

Supplement: Supplementary file 1 — Figure S1. Correlations of MRI‐ and BIA‐based measurements of skeletal muscle mass in non‐obese (light circle) and obese (dark triangle) subjects. Figure S2. Correlations of AMMITotal (A) and AMMIFat‐free (B) with age, BMI, VAT and physical activity. Table S1. Associations between demographics, cardiometabolic risk factors and AMMITotal and AMMIFat‐free Table S2. Obesity‐stratified associations of MRI‐ and BIA‐based measurements of skeletal muscle muss. [file JCSM-13-976-s001.zip › JCSM_12913_SuppFig2B.jpeg]
